# Multi-Phase Flow Metering in Offshore Oil and Gas Transportation Pipelines: Trends and Perspectives

**DOI:** 10.3390/s19092184

**Published:** 2019-05-11

**Authors:** Lærke Skov Hansen, Simon Pedersen, Petar Durdevic

**Affiliations:** Department of Energy Technology, Aalborg University, 6700 Esbjerg Campus, Denmark; lsh@et.aau.dk (L.S.H.); pdl@et.aau.dk (P.D.)

**Keywords:** multi-phase flow, offshore, oil and gas, flow metering, instrumentation

## Abstract

Multi-phase flow meters are of huge importance to the offshore oil and gas industry. Unreliable measurements can lead to many disadvantages and even wrong decision-making. It is especially important for mature reservoirs as the gas volume fraction and water cut is increasing during the lifetime of a well. Hence, it is essential to accurately monitor the multi-phase flow of oil, water and gas inside the transportation pipelines. The objective of this review paper is to present the current trends and technologies within multi-phase flow measurements and to introduce the most promising methods based on parameters such as accuracy, footprint, safety, maintenance and calibration. Typical meters, such as tomography, gamma densitometry and virtual flow meters are described and compared based on their performance with respect to multi-phase flow measurements. Both experimental prototypes and commercial solutions are presented and evaluated. For a non-intrusive, non-invasive and inexpensive meter solution, this review paper predicts a progress for virtual flow meters in the near future. The application of multi-phase flows meters are expected to further expand in the future as fields are maturing, thus, efficient utilization of existing fields are in focus, to decide if a field is still financially profitable.

## 1. Introduction

**Figure 1:**
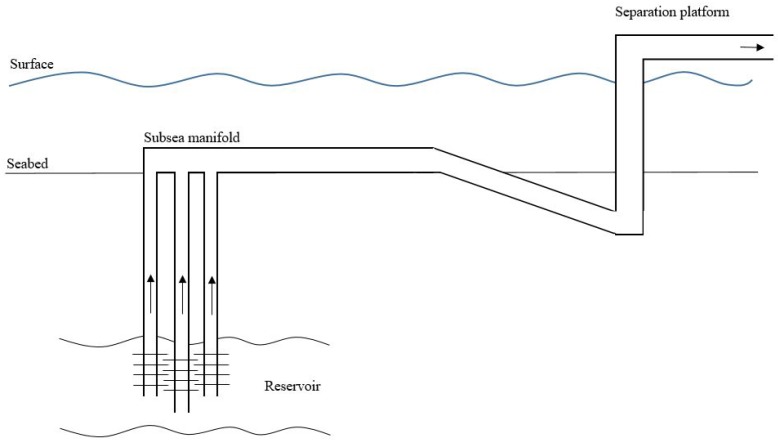
Subsea manifold and transportation pipelines to separation platform.

One major problem in the offshore oil and gas industry is monitoring of multi-phase flow consisting of oil, water and gas in pipelines [1,2]. Due to difficulties regarding subsurface instrumentation the multi-phase flow contributes to a huge problem at offshore installations [3,4]. In Figure 1a typical offshore installation of a well-pipeline-riser system is illustrated. As it can be seen from the figure, most of the process is placed subsurface, which enhances the problem. For vertical wells as in Figure 1 the system consists of three main sections being the vertical pipe from the reservoir to the seabed, the horizontal subsea pipeline, and the vertical riser-pipeline from the seabed to the separation platform [5]. Since subsea instrumentation is extremely expensive and cumbersome, monitoring of the multi-phase flow is often reduced to the top of the vertical riser-pipeline and following pipelines, which is located above sea.

Poor measurements of the multi-phase flow can lead to big uncertainties regarding important data, which due to small measurement errors and round-offs can end up with a huge error margin in the end of the oil recovery process [6]. The problem is well-described and investigated, but the potential errors in the measurements are often not being accounted for during the oil recovery process, and a lot of models and empirical algorithms can hereby be questioned upon their accuracy. Poor accuracy of multi-phase flow measurements can have a huge effect on:

- Model prediction, history matching and future of reservoir [7,8,9,10,11,12].
- Control of flow patterns [13,14].
- Separation [15].
- Chemical injection [16].
- Emulsion layer [16].
- Corrosion-rate [17].

Although the focus of this review paper is multi-phase flow in the upstream transportation pipelines, the flow measurement is also of importance to other parts of the oil recovery process. Tthe produced water (PW) treatment is affected by accurate and reliable measurements of the flow. PW treatment is a product from the separation process, which occurs after Figure 1 on the separation platform. It has been documented that control of flow patterns (e.g., slugs) can reduce the separation efficiency of the separator, and hereby optimize the PW treatment process [18,19,20]. Slugs can be controlled by a feedback system feed with flow measurements from e.g., a multi-phase flow meter (MPFM).

**Figure 2:**
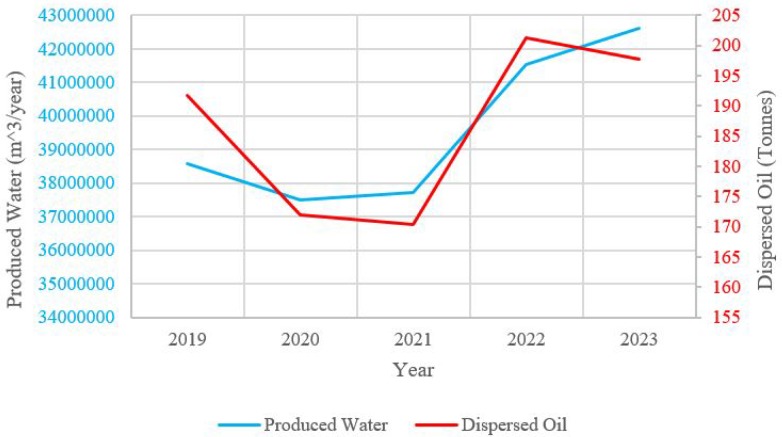
Produced water and discharged dispersed oil from the Danish platforms in the North Sea. The platforms include: Dan, Gorm, Halfdan, Tyra, Syd Arne and Siri. The fields are operated by Total E&P Danmark A/S, Hess Danmark ApS and INEOS Oil & Gas. The blue graph illustrates the total amount of produced water from the fields. The red graph illustrates the discharged dispersed oil in the PW.

The Danish Environmental Protection Agency has specified regulations on the PW with respect to dispersed oil discharged into the ocean [21,22,23]. All Danish platforms in the North Sea are by law required to discharge less than 222 ton of oil per year in total [21,24]. The regulations are stated by The Danish Environmental Protection Agency based on requirements from OSPAR Commission, which protects the marine environment and biodiversity in the North-East Atlantic Ocean [25]. The amount of dispersed oil in PW can carefully be monitored and hereby reduced by the implementation of e.g., MPFMs, as they can optimize the separation process and chemical injection. In the permissions provided by The Danish Environmental Protection Agency given from 2019 to 2023 it can be seen from Figure 2 that the amount of PW and discharged dispersed oil is increasing with exception of some years due to shut down of big platforms (reconstruction of Tyra field in 2019–2022 [26,27]). The increase of PW is a result of maturing fields with increasing water cut. This is supporting the importance of accurate measurements of the multi-phase flow and, hereby, the necessity of MPFMs. The increase in PW can also be a huge problem, if the legal requirements are not adjusted to this change, as it will be difficult to respect the given law. The data from Figure 2 are based on reports from The Danish Environmental Protection Agency regarding discharge permissions of dispersed oil in PW for the Danish North Sea fields operated by Total E&P Danmark A/S, Hess Danmark ApS and INEOS Oil & Gas [21,22,23].

This article will cover monitoring of multi-phase flow in the main production upstream in the 1st stage separation process. It will evaluate the respective methods based on how they can be implemented and applied to benefit the entire oil recovery process. First, an introduction to the problem will be described. Section 1.1, Section 1.2 and Section 1.3 will introduce the most crucial issues with unreliable and inaccurate multi-phase flow measurements. Section 2 will explain the conventional method for measuring multi-phase flow, where Section 3 will describe the existing technologies and present some non-commercial prototypes. After an introduction to current technologies, some industrial and commercial MPFMs will be presented and compared in Section 4. Lastly, a discussion and conclusion will be made in Section 5 and Section 6, evaluating and predicting the future of MPFMs based on the results obtained in this study.

### 1.1. Model Prediction, History Matching and Future of Reservoir

With an accurate model of the reservoir, it is possible to approximate the future behavior of the field, to perform computer simulations and to manage the reservoir. The model is periodically updated based on observed reservoir behavior, which is based on e.g., multi-phase flow measurements of the wells. The approach is called history matching and has been described in [7,8,9,10,11,12], where possible optimization options are also introduced. History matching is an approach where the current reservoir model is fitted to reproduce the past behavior, so that the oil recovery is at its maximum over the lifetime of the field. This can e.g., be done by enhanced oil recovery process [28,29,30,31]. To see the behavior of the model and the oil production, decline curve analysis is employed. Decline curve analysis is applied for production forecasting and reserves estimation [32]. The decline curve analysis is introduced in [33,34,35], and based on the decline of the curve, enhanced oil recovery methods such as water injection are decided and implemented. The data for the decline curve analysis is based on performance history and observed production over time. These datasets, which e.g., consist of the phase fraction and velocity of the multi-phase flow, need to be accurate to avoid errors in the model. Hence, the water decision of injection and injection volumes is dependent on the accuracy of the multi-phase flow measurements of the wells.

### 1.2. Flow Regimes

Many studies investigate the flow pattern inside the transportation pipelines [36,37,38,39,40]. Especially three-phase flow of water, oil and gas can be a huge challenge in the oil and gas industry [41]. There are several factors that can affect the liquid–gas flow pattern, which has been stated in [42] and is listed below:

- Phase properties, fractions and velocities.
- Operating pressure and temperature.
- Diameter, shape, inclination and roughness of the transportation pipe.
- Presence of e.g., valves, T-junctions and bends.
- Pipe direction: vertical, horizontal or incline/decline.
- Type of the flow: whether the flow is in steady-state, pseudo steady state or unsteady (transient).

There are several types of flow patterns such as bubble, churn, annular, disperse and slug flow [43,44,45,46]. The latter can be difficult to monitor and prevent, and requires an accurate feedback system with reliable measurements, in order to prevent slugging from occurring inside the pipes. The input for the feedback system can e.g., be multi-phase flow or pressure measurements. It is proven that flow measurements are a better control variable compared to e.g., pressure, as long as the flow measurements are accurate. The effectiveness of a cascade controller, which is i.a. based on flow measurements, is presented in [47]. This is supporting the importance of reliable and accurate flow measurements provided by e.g., MPFMs.

### 1.3. Separation and Chemical Injection

In the gravity separator the three phases will start separating right after entering the gravity separator. Small impurities from the well and corrosion inhibitors added to the flow can lead to the presence of foam between the oil and gas mixture [16,48]. In addition to the foam between the oil and gas phases there will also occur an emulsion layer between the oil and water phases. The thickness of the emulsion layer depends on i.a. the residence time inside the gravity separator [48]. Both the foam and emulsion layer has a negative impact of the separation process and hereby the total oil recovery [16]. Chemicals are added to prevent the foam and thick emulsion layer inside the separator. The amount of chemicals added depends on the composition of the fluid, and accurate measurements of the multi-phase flow are hereby essential. The chemical injection is a very expensive process and unnecessary injection will only lead to poor oil quality and unnecessary expenses [49]. Hence, the need for measurement of all three phases prior separation is necessary. Another way to prevent a thick emulsion layer between the oil and water is electrostatic coalescence, which has been described in [50,51,52]. The stream of the multi-phase flow inside a pipeline can also be directly connected to corrosion [53]. In the presence of slug flow conditions as explained in Section 1.2, the multi-phase flow has the impact of increasing the protective surface scales/films inside the pipeline. This can eventually increase the corrosion rate since the slug flow will lead to higher fluctuations of the surface shear stress [54]. Each problems can be prevented by obtaining correct measurements of the multi-phase flow, which will be an input in a feed-back system. Reliable and accurate equipment is essential to monitor the multi-phase flow and hereby to improve the overall oil recovery.

## 2. Conventional Flow Measurement Technology

**Figure 3:**
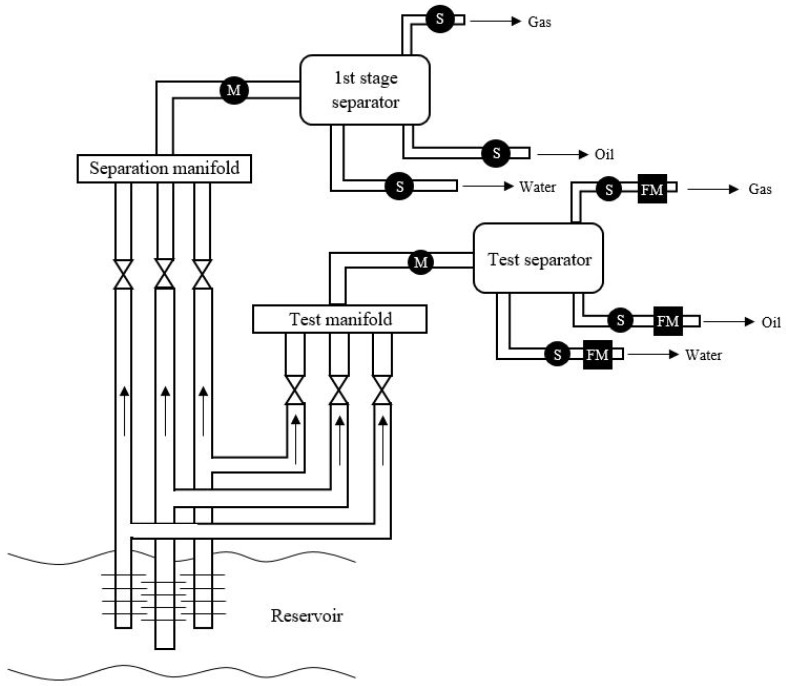
Oil production system with test separator and 1st stage separator. The flow inside the pipe is denoted as either M for multi-phase flow or S for single phase flow. After the test separator each phase flow is ideally measured by a single-phase flow meter (FM).

This section will provide the reader with an introduction to the conventional technology to measure and monitor the multi-phase flow. The produced multi-phase flow of a reservoir can vary depending on the location and lifetime of the well [55]. Previously the multi-phase flow has been measured using a test gravity separator, which measured each single phase flow from the output [42,56,57]. Commonly used meters for single-phase flow measurements can e.g., be a venturi meter, turbine meters or coriolis flow meters [1,58]. The conventional flow technology is illustrated in Figure 3. If the fraction or velocity of each phases is to be obtained, the multi-phase flow can be transported to a test platform, where the test separator is located.

Inside the test separator the multi-phase flow is separated into three different single-phase flows. After each outlet of respectively oil, gas and water a single-phase flow meter is installed labeled as FM on Figure 3. The conventional method with the test separator is reliable and accurate but not suitable for real-time monitoring of the multi-phase flow, as the process is simply too slow [30]. It takes time for the three phases to divide inside the separator, which means that the single-phase output will not provide instantaneous and real-time measurements [59]. Since the measurements are not in real-time it can not be used in a feedback system to prevent e.g., slugging or overdosage of chemical injection. Other disadvantages regarding the conventional technology is that the test separator has a huge footprint and contributes to extra load on the given platform [42]. Also the measurements are not performed in-line [59]. Due to these disadvantages regarding the conventional test separator, new technologies and instruments have been investigated and implemented for commercial use. The upcoming sections will cover the new technologies to measure the multi-phase flow.

## 3. Multi-Phase Flow Metering

Generally when measuring the multi-phase flow the mass and volumetric flow rates of water, oil and gas need to be obtained. As described in [6] Equation (1) is valid for the volumetric flow rate *Q*.

(1) $$Q=A(\alpha v_{g}+\beta v_{w}+\chi v_{o})$$

A is the cross-section area of the pipe. α, β and χ are the gas void fraction, water fraction and oil fraction respectively. $\;v_{g}$, $\;v_{w}$ and $\;v_{o}$ is the instantaneous velocity of gas, water and oil. The sum of the fraction of the three phases should equal one, which means that only two of the three phase fractions need to be measured. Equation (1) can then be simplified as Equation (2):

(2) $$Q=A(\alpha v_{g}+\beta v_{w}+\left[1-\left(\alpha +\beta \right)\right]v_{o})$$

To calculate the mass flow rateM of the multi-phase flow, the density of each phase needs to be obtained. The mass flow rate is calculated in Equation (3):

(3) $$M=A(\alpha v_{g}\rho_{g}+\beta v_{w}\rho_{w}+\left[1-\left(\alpha +\beta \right)\right]v_{o}\rho_{o})$$

where $\rho_{g}$, $\rho_{w}$ and $\rho_{o}$ is the density of the gas, water and oil [6]. To monitor the volumetric and/or mass flow rate of the multi-phase flow, different technologies have been invented. Over a lifetime of the well each of the parameter can vary. Hence, it is important for the MPFM to measure both density, velocity and phase fraction of the flow.

Some of the most common methods will be introduced in the next sections. For a technology to be sufficient for multi-phase flow measurement, it should be non-intrusive, flow regime independent, accurate, reliable and able to measure the entire fraction range of each of the phases [55]. The upcoming technologies will be discussed on their ability to monitor the multi-phase flow. Section 3.1.1 examines electrical capacitance tomography, Section 3.1.2 examines electrical resistance tomography, Section 3.1.3 examines electromagnetic tomography, Section 3.1.4 examines microwave tomography, Section 3.1.5 examines electrical impedance tomography and lastly Section 3.1.6 examines optical tomography. Section 3.2 will introduce a different technology called gamma densitometry. Section 3.3 will introduce a technology called virtual flow meters (VFM), which consists of i.a. differential pressure transmitters such as an orifice plate or venturi meter.

### 3.1. Tomography

Tomography is an imaging process technology, which is widely used when measuring the multi-phase flow in the offshore industry [55,60,61,62,63,64,65,66,67,68,69,70]. The advantages of tomography is that the sensors are placed at the periphery of the pipe and are hereby not a causing any obstruction to the flow [71]. Equation (4) is applied to determine the volume flow of a phase [56]:

(4) $$Q_{x}=a\sum_{i=1}^{N}f_{i(x)}v_{i}$$

**Figure 4:**
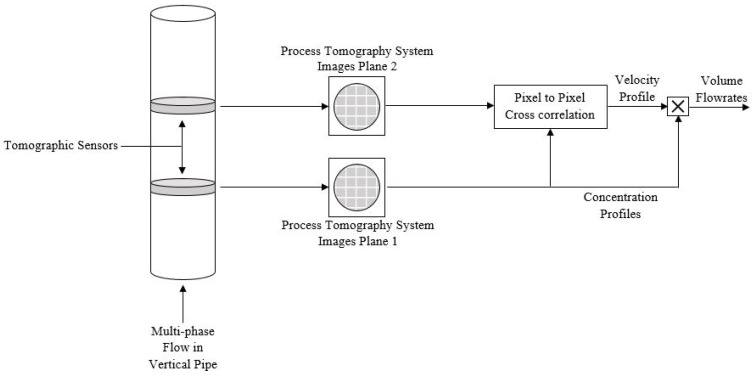
Multi-phase flow measurement using tomography imaging process.

$f_{i}$ is the volume fraction of phasex and$\;v_{i}$ is the flow velocity in thei-th element.N is the elements that the cross-section of the pipe is divided into with an equal areaa. The tomography technology is illustrated in Figure 4, where two series of images are taken simultaneously. Each series are representing a cross-section of the pipe, which are cross-correlated to obtain the velocity profile$\;v_{i}$. The volume fraction distribution of the phase$\;f_{i(x)}$ can be obtained directly by the images provided from the tomographic sensors [56].

#### 3.1.1. Electrical Capacitance Tomography (ECT)

**Figure 5:**
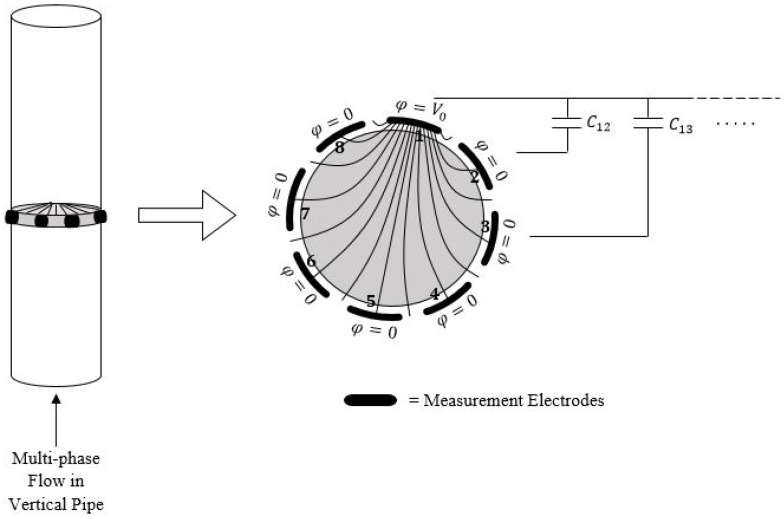
ECT sensor with 8 electrodes around the pipe. One electrode is excited and the capacitance is measured.

Electric capacitance tomography has been used for many years and the technology has been widely investigated [72,73,74,75,76,77,78]. In the electrical capacitance tomography a multi-electrode sensor obtains capacitance measurements. The electrodes are located peripherally around the pipe causing no interruption with the flow. For an ECT sensor the capacitance is changing when the di-electric material distribution is changing [56,79]. The technology for an electrical capacitance tomography sensor is illustrated in Figure 5. A typical ECT sensor consist of between 8, 12 and 16 measurement electrodes [56,80].

One issue regarding ECT is the imaging reconstruction algorithm, which induces an inverse problem. Different approximation methods have been used to solve the inverse problem and linear back projection (LBP) is commonly used. Reference [81] has developed a new reconstruction algorithm, which they claim is able to image two- and three-phase flows. The algorithm is based on an analog neural network multi-criteria optimization image reconstruction technique and shows both accurate, consistent and robust results. The algorithm works for transient multi-phase phenomena in gas-liquid and gas-liquid-solid flows. Some non-commercial ECT techniques have been proposed, where [82] presents a void fraction measurement system for two-phase flow. The measurement error of the system is less than 5%, and the method is suitable for the void fraction measurement of bubble flow, stratified flow, wavy flow, slug flow, and annular flow. Another promising non-commercial technique is presented in [83]. The prototype is a multi-phase flow meter for oil-continuous flows. The technique is an improved AC-based ECT system and it shows less than 3% absolute error for oil-water flows with a water liquid ratio (WLR) < 35%.

#### 3.1.2. Electrical Resistance Tomography (ERT)

ERT is contrary to ECT applied when the continuous phase is conducting [56]. It can be a challenge that the continuous phase needs to be conducting, since the phases can vary e.g., during a slug cycle. During a slug cycle the gas volume fraction (GVF) can vary from 0–100% and the continuous phase is hereby not guaranteed to be conducting at all times. ERT has also been widely described and investigated [84,85,86,87,88]. The electrodes of an ERT sensor are located in direct contact with the flow inside the pipe.

The ERT technology is only suitable for measurements in vertical pipes, since the electrodes are in direct contact with the flow. If the electrodes are frequently exposed to the gas phase (non-conducting phase), as will happen in horizontal pipes under stratified, wave, slug or plug flow, the electrodes might lose their continuous electrical contact with the measured flow [89]. In [89] a method is presented, so ERT can be implemented for two-phase flow in horizontal pipes. The method is called Liquid Level Detection and takes account for the electrodes that are exposed to gas (air) and hereby monitors the position of the water surface. M. Wang [90] has also invented a method for ERT sensors to address the challenges of electrodes with no contact to the conductive fluid. A disadvantage regarding ERT is that the technology is primarily suitable when the continuous phase is conducting. Therefore, when the flow is water continuous, ERT should be applied. This is due to the electrical conductivity of water compared to oil, which will appear as an insulator [91]. Due to this it can be beneficial to combine the ERT and ECT technology, to obtain a sensor technique that can obtain capacitive and resistive properties simultaneously [92]. The design of a multi-modal tomography system based on ERT and ECT has been described in [93,94,95,96]. In [97] a dual-modal sensor is presented, which is able to measure the multi-phase distribution in a flow. For a gas-oil-water concentration consisting of 50% oil/water (30% water and 20% oil), the sensor is able to reconstruct the images such that the concentration is calculated to 49.69% oil/water (30.17% water and 18.53% oil). The ECT mode is used when WLR is less than 40% (oil-continuous flows) and ERT is used when WLR is higher than 40% (water-continuous flows).

#### 3.1.3. Electromagnetic Tomography (EMT)

Electromagnetic waves use the permittivity of a fluid to determine the fraction of each phase in a multi-phase flow [98]. The sensor consists of excitation coils, which produce a magnetic field. The sensors are not in direct contact with the flow [56]. Water has a higher permittivity than oil and gas and the sensor is therefore, more sensitive to water flows. The permittivity is represented with the symbol epsilon. Water has a permittivity at $\varepsilon_{r(water)}$ = 81, oil at $\varepsilon_{r(oil)}$ = 2.2–2.5 and gas at $\varepsilon_{r(gas)}$ = 1 [98]. The EMT sensor is not the most convenient technology, when it comes to monitoring of multi-phase flow in the offshore industry. Since the measurements are based on the electrical conductivity and the magnetic permeability of the medium, it will require a high excitation frequency to increase the signal from the sensor [99]. In a recent research [100] a combination of magnetic induction tomography (MIT) and electromagnetic velocity tomography (EVT) showed promising results in measuring the velocity of the continuous phase (water) in a two-phase flow consisting of oil and water. MIT and EVT are both types of electromagnetic tomography techniques. The prototype shows great accuracy for single-phase water flow with a relative error of only 0.012%, but lacks in accuracy with a ratio of 65.80% water in water-in-liquid multi-phase flow. Here the relative error is 12%, which is a huge error-margin, if the meter should be implemented for multi-phase flow measurements.

#### 3.1.4. Microwave Tomography (MWT)

One way to obtain electromagnetic waves is by using microwave tomography [98,101,102]. A microwave tomographic sensor consists of both receiving and transmitting antennas [103]. By using an electromagnetic field the electromagnetic waves will be transmitted at different angles and hereby create an image of the flow inside the pipe, while comparing with an uniform permittivity background at the receiving part [77,103]. The hardware of a microwave sensor consists of a source that generates the microwave signals, a detection part that detects and measures the microwaves, a routing part that converts the signals into multi-views (images) of the flow and lastly microwave transmitting and receiving antennas [77]. The microwave tomography technology is not widely used for multi-phase flow measurements due to the image reconstruction algorithm, as this is too slow for real-time imaging of the dynamic behavior of the multi-phase flow [103]. An experimental MWT system is presented in [104], where an 8-port sensor is designed for oil-gas-water flows. The image construction is not yet accurate enough to be implemented for industrial and commercial application, as the image quality and hereby meter accuracy decreases with increasing frequency. Further investigation is essential to present an accurate MWT meter for multi-phase flow measurements at offshore installations.

#### 3.1.5. Electrical Impedance Tomography (EIT)

Electrical impedance has been described in [105,106]. This technology is often used in the pharmaceutical industry to test respiratory and lung function [107]. The electrodes are located periphery around the pipe and have electrical contact with the flow inside the pipe but do not cause any obstruction to the flow [80]. A current is injected through the cross-section of the pipe and the corresponding electrode voltage is measured. To calculate the fraction of each phase an algorithm is used. The input to the algorithm is the applied current pattern and the electrode voltages, which will then reconstruct an image based on the electrical conductivity and permittivity of the flow [108]. EIT is not widely used in the oil and gas industry, but [105] shows promising results with a recently developed measurement system that can produce real time 3D images. These are to be used together with an algorithm to monitor the multi-phase flow. The system consists of 80 surface electrodes and is capable of producing 15 frames per second in 3D. Another EIT sensor described in [109] presents a multi-mode prototype, with a combination of capacitive and resistive/conductive mode. By experiments the relative errors between measured and calculated values are below 1.64% for the capacitance mode and less than 2.68% for the conductive mode. The sensor requires further development before application but indicates promising results for measurements of multi-phase flow.

#### 3.1.6. Optical Tomography

Optical tomography uses illumination such as absorption, diffraction and reflection of light as a method to measure the multi-phase flow within a cross-section of a pipe [60,110]. The main component of an optic sensor is a light source and a camera to sense the reflected light. Since the sensor is measuring the transparency of the flow inside the pipe with respect to absorbed and reflected light, the sensor needs a transparent window inside the pipe in order to be able to detect the light [111]. Another disadvantages regarding optical tomography is that in multi-component flow such as multi-phase flows, a bubble of e.g., air/gas can cause misleading measurements. This is due to the curving and reflecting surface of the bubble, which can cause an intense beam of light to be reflected within the flow and hereby confuse the camera of the optic sensor [80]. Optical tomography technology has been described in [112], where the sensor is investigated for both single and two-phase pipe flows. The sensor shows accurate measurements but are limited to flow situations with up to 15% gas fraction. The lack of ability to measure the gas fraction in the entire range makes the optical tomography not suitable for monitoring of multi-phase flow in the oil and gas industry.

### 3.2. Gamma Densitometry

Besides tomography another convenient technology is gamma densitometry. Gamma densitometry uses a radioactive source to obtain measurements of the multi-phase flow. Gamma is preferred because of its ability to measure spacial distribution based on the atomic number and density of a material [111,113,114]. When gamma-rays are radiated from the source to the detector, the ray will attenuate depending on the absorption of radiation of the flow within the pipe [115]. Depending on the detected gamma quanta at the detector, the sensor is able to measure even small changes in the density differences of the fluid, and can hereby obtain accurate measurements of the fraction of each phases [116]. To measure both the phase volume fraction and velocities, and not only the average fluid density, the gamma densitometer is often installed together with an equipment such as e.g., a venturi meter or an orifice plate. In [117] researchers presents a single clamp-on gamma densitometer unit, which is able to determine both phase volume fractions and velocities to predict the individual phase flow rates of vertically upward multi-phase flows. The method yield promising improvements on the accuracy, but still needs more investigation as the densitometer is flow dependent. Gamma densitometry has also been tested and evaluated in [118,119,120]. A disadvantage with gamma-rays is the salinity content in the water. Saltwater has a higher attenuation coefficient than freshwater, which will cause errors in the measurements if the salinity content changes [98]. To avoid these errors additional equipment is needed. A single-beam gamma densitometer with an accuracy of 0.97% (phase fraction measurements) is presented in [121]. The applied gamma source is Am-241, with radiation energy of 59.5 keV. The accuracy of the desitometer is increased by increasing the measuring time and the location of the radioactive source with respect to the pipe. With increased measuring time and the radioactive source at the center of the pipe, an accuracy of the phase fraction measurements on 0.53% can be achieved. Another method for detecting the flow regime and void fraction by the use of a gamma source is presented in [122,123]. The method is based on dual modality densitometry using artificial neural network (ANN) and presents error less than 1% between estimated and simulated values.

### 3.3. Differential Pressure Meters

Many multi-phase flow meters use differential pressure (DP) transmitters to measure the difference in the pressure in two given points inside the pipe. The most common differential pressure transmitters used in the offshore industry are an orifice plate or a venturi meter, due to their accuracy, in-line measurements and small footprint. Using Bernoulli’s principle it is known that increasing the velocity of the fluid will cause a decrease in the pressure. By obtaining the differential pressure, the flow rate of the fluid can be calculated [124,125]. Both the orifice plate and the venturi meter creates a disturbance to the flow which enables the DP transmitters to measure the pressure difference between two points. Together with a software tool consisting of empirical algorithms, some DP meters can provide multi-phase flow measurement with the same accuracy as tomography based meters or gamma densitometers. These meters are called virtual flow meters (VFM) and with the simple instrumental equipment, these meters can contribute to a cheaper solution for the offshore industry [126]. The meter consist only of an orifice plate or a venturi meter and already available measurements at offshore installations such as temperature and pressure transmitters. The main limitation of the meter is that the fluid composition must be constant. To avoid this problem void fraction sensors and gamma densitometers are combined with the VFM measurements.

#### 3.3.1. Orifice Plate

**Figure 6:**
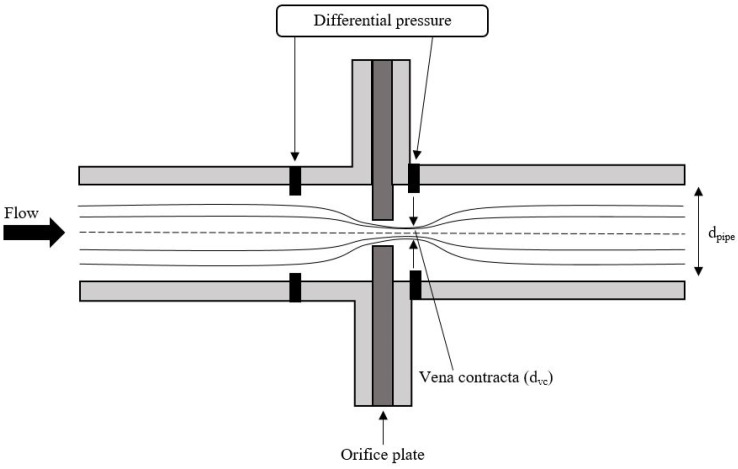
Principle of an orifice plate. Interruption of the flow inside a pipe due to an orifice plate. DP transmitters are measuring the pressure difference at a point before and after the orifice plate and the velocity of the fluid is hereby obtained by Bernoulli’s equation.

Orifice plates can be used to measure the flow velocity of a fluid within a pipe [127,128,129]. As explained in [130] an orifice plate works by applying a thin plate with a small opening inside the pipe. The orifice plate is illustrated in Figure 6.

The point where the flow is experiencing the maximum of convergence is called vena contracta. Vena contracta is occurring just after the orifice plate as illustrated on Figure 6 with the diameter noted as$\;d_{vc}$. The differential pressure transmitters measures the pressure in the regular pipe diameter ($d_{pipe}$) and at vena contracta and hereby calculates the pressure difference and by Bernoulli’s equation obtains the velocity of the fluid.

For vertical orifice plates the volumetric flow rate in terms of the pressure difference (ΔP) is calculated as Equation (5):

(5) $$Q=C_{d}A_{ori}Y\sqrt{\frac{2(\Delta P+\rho g\Delta z)}{\rho (1-\beta_{d}^{4})}}l$$

$\beta_{d}$ is the ratio between the diameter of the pipe and the diameter of the orifice,z is the change in elevation and $C_{d}$ is the discharge coefficient. $A_{ori}$ is the area of the orifice plate, ρ is the density of the fluid andg is gravity. The discharge coefficient for an orifice plate (thin sharp edged) is around 0.61 [131]. *Y* is the expansion coefficient, which is defined as Equation (6):

(6) $$Y=\frac{C_{d,c}}{C_{d,i}}$$

*Y* depends on the discharge coefficient for compressible ($C_{d,c}$) and incompressible ($C_{d,i}$) flows [132]. For incompressible fluids *Y* = 1 and for compressible fluids the expansion coefficient will be defined by the discharge coefficients as defined in Equation (6). The orifice is often used in a combination with another instrument to measure and calculate the mass flow rate of the multi-phase flow. An orifice plate meter is presented in [126]. The measurements of the meter are compared with simultaneously measured data from a test separator and shows 3.52% measurement error with respect to the standard volume flow rates of oil, water and gas.

#### 3.3.2. Venturi Meter

**Figure 7:**
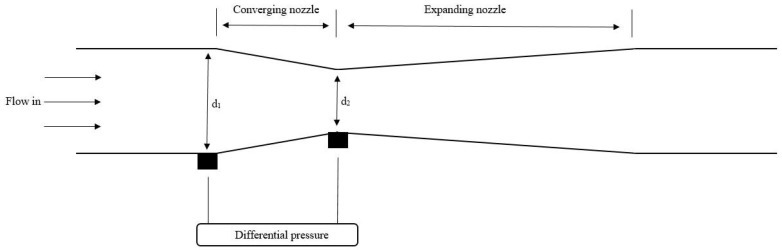
Principle of a venturi meter. The DP transmitters are located before the pipe is converging ($d_{1}$) and when the pipe is most converged ($d_{2}$).

A lot of studies has shown the effectiveness of measuring the multi-phase flow by using a venturi meter [125,133,134,135,136,137]. The principle of a venturi meter is common to the principle of an orifice plate and illustrated in Figure 7. A venturi meter is said to have the lowest pressure loss compared to other differential pressure transmitters [65].

To obtain the volumetric flow rate using a venturi meter, the same equation as the orifice plate is used. The only difference in Equation (5) is that the discharge coefficient is higher for a venturi meter compared to the orifice plate. For a venturi meter the discharged coefficient ($C_{d}$) is between 0.984–0.995 [131]. The venturi meter is widely used with respect to multi-phase flow in the offshore industry. In [65] a solution has been presented for a two-phase flow meter consisting of an ECT sensor and a venturi meter. The ECT sensor is measuring the void fraction information’s while the venturi meter is obtaining the velocity of the two-phase flow. The venturi meter has also been investigated together with an ERT sensor. This is described in [137], where the method is presented. The ERT sensor measures the real-time flow pattern, while the void fraction and mass quality is calculated and determined by the presented model. The mass flowrates are calculated based on the mass quality and the differential pressure across the venturi meter. For bubble and slug flow the root mean square error of the total mass flowrate is less than 0.03 and 0.06 respectively. The relative error is less than 5%.

### 3.4. Wet Gas

When the flow inside the pipe is gas dominant and the water and oil fraction together is less than 5%, wet gas conditions are valid [6,138]. A wet gas flow can be defined by Lockhart-Martinelli parameter, which is a dimensionless number ranging from 0 to 0.3. Zero is representing a completely dry gas [139]. As described in [140] the Lockhart-Martinelli parameter can be defined as Equation (7).

(7) $$X_{LM}=\sqrt{\frac{SuperficialLiquidInertia}{SuperficialGasInertia}}=\frac{m_{l}}{m_{g}}\sqrt{\frac{\rho_{g}}{\rho_{l}}}$$

where $m_{l}$ is the mass flow rate of the liquid and $m_{g}$ is the mass flow rate of the gas. $\rho_{g}$ and $\rho_{l}$ is the density of gas and liquid respectively. To predict the wet gas flow pattern the Lockhart-Martinelli parameter combined with the gas and liquid densimetric Froude number is used [140]. The gas and liquid densimetric Froude number is defined as Equations (8) and (9) [140]:

(8) $$Fr_{g}=\frac{{}^{U_{sg}}}{\sqrt{gD}}\sqrt{\frac{\rho_{g}}{\rho_{l}-\rho_{g}}}$$

(9) $$Fr_{l}=\frac{{}^{U_{sl}}}{\sqrt{gD}}\sqrt{\frac{\rho_{l}}{\rho_{l}-\rho_{g}}}$$

*D* is the internal diameter of the pipe, *g* is the gravitational constant, $\rho_{g}$ and $\rho_{l}$ is the densities of gas and liquid, and $U_{sg}$ and $U_{sl}$ is the superficial gas an liquid velocities calculated by Equations (10) and (11).

(10) $$U_{sg}=\frac{m_{g}}{\rho_{g}A}$$

(11) $$U_{sl}=\frac{m_{l}}{\rho_{l}A}$$

$m_{g}$ and $m_{l}$ is the mass flow of gas and liquid. Wet gas flow meters can consist of i.e., an orifice plate, which has been described in [140] or a venturi meter [138].

### 3.5. Summary of Current Technologies

Section 3.1, Section 3.2, Section 3.3 and Section 3.4 has outlined some of the current technologies for multi-phase flow measurements. Based on the investigation and experimental results especially ERT, EIT and gamma densitometry have shown promising and reliable results based on the accuracy of the presented prototypes. All of the presented prototypes are listed in Table 1 for a better overview. Though, the prototypes has been widely tested, further investigations should be done before application of the prototypes for industrial use in e.g., the offshore industry. The prototypes with the poorest accuracy is the VFMs by [65,126,137]. The ECT meter from [82] shows a measurement error on 5%, which is higher than some of the other presented prototypes. The most accurate prototypes are the two MPFMs with a radioactive source. Reference [121] shows less than 0.53% measurement error, while [122,123] shows less than 1% mean absolute error.

## 4. Comparison of Industrial MPFMs

In this section some industrial MPFMs will be presented. They will be listed based on i.a. their technology, advantages and disadvantages and will be illustrated in Table 2. Please note that the tabular only represents a small amount of the existing commercial MPFMs on the market. It gives a small insight to the industry and which technologies the industry has implemented. It should also be noted that the values given in the table is based on data sheets provided by the manufactures and that it has not been possible to verify the data.

From the table it can be seen that the presented MPFMs with a radioactive source tends to have a better accuracy compared to the MPFMs with no radioactive source. The MPFMs provided by Emerson (Roxar) 2600 M and 2600 MG contains no radioactive source and has a poor accuracy compared to the other MPFMs. Also the MPFM provided by Khrone Oil & Gas contains no radioactive source and has a poor accuracy especially for the gas rate. Based on the data sheets the most accurate MPFMs from the table are the meters provided by Schlumberger, Weatherford and Pietro Fiorentini (Flowatch HS). The three meters all contain a radioactive source and is claimed to operate in the entire range (0–100% WLR, 0–100% GVF ).

## 5. Discussion of Advantages and Disadvantages Regarding MPFMs

The next sections will cover a discussion of the instrumental perspective of MPFMs based on the cost, maintenance, footprint, radioactive source and calibration. This will contribute to the conclusion and the future predictions for the MPFMs.

### 5.1. Cost

The price for a MPFM can vary between 100,000–500,000 US dollars, depending on the requirements for the meter [42]. The prices will vary depending on whether the MPFM is installed on- or offshore or if the location is topside or subsea. The operational cost for a MPFM is around 10,000–40,000 US dollars per year. This is a huge saving compared to the conventional test separator, which has an operational cost around 350,000 US dollars per year [42]. It is clear that VFMs are much cheaper compared to other MPFMs, as they only consist of measurements based on simple conventional field instruments and empirical algorithms.

### 5.2. Maintenance

Whether the MPFM consists of pressure and temperature sensors, gamma-ray source etc. can have an influence of the maintenance of the instrument. Maintenance is always a cumbersome procedure when it is performed offshore. If maintenance is performed on an unmanned platform, the procedure can be challenging and expensive due to shipping of the right personnel to the platform. Another contributing factor to the expenses of maintenance is whether the equipment i located sub sea or above sea level. If the equipment is located sub sea the maintenance is assigned specially educated divers and consultants, which is a cumbersome and expensive procedure [158,159].

**Figure 8:**
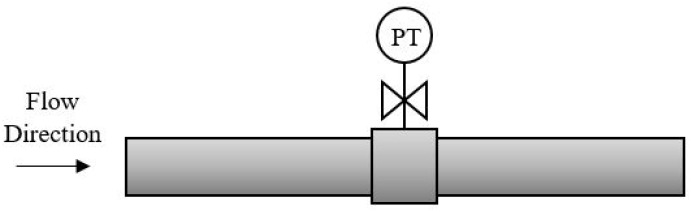
A PT sensor placed on a pipe. The PT sensor is replaced without causing any affection on the oil production due to the location of the valve.

**Figure 9:**
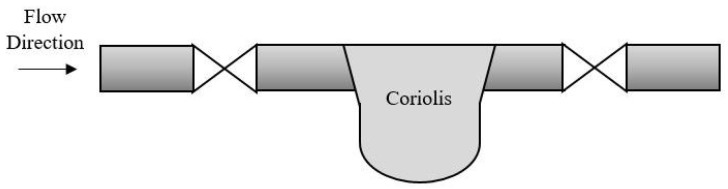
A coriolis meter placed inline of the pipe. Replacement of the meter will cause a shut down in the oil production in the given location.

Pressure and temperature sensors are installed so that they can easily be adjusted or replaced by new sensors, which makes maintenance more doable. As illustrated in Figure 8, a sensor is measuring either the pressure or temperature on a fluid flowing inside a pipe. The valve can easily be shut down without any interruption of the fluid, and the sensor can be replaced without a complete shut down of the oil production. If the MPFM receives the mass flow and density distribution from a coriolis, the maintenance and replacement can cause more difficulties [160,161]. Even though the coriolis is suppose to have lower maintenance due to no moving parts, it can still cause problems. The coriolis is installed inline of the pipe as illustrated in Figure 9, and maintenance such as replacement of the coriolis will require a complete shutdown of the production.

Maintenance of a gamma source is not required often. The radiation of the source will decay over time with respect to the half-life time of the radioactive source. If a gamma source is to be maintained or replaced, the procedure is expensive as specialized and certificated personnel is shipped to the concerned platform.

### 5.3. Footprint

The footprint of the equipment is essential at offshore installations. A compact platform makes huge constructions and equipment impossible and the footprint of a MPFM should be as small as possible. Therefore a solution with an inline MPFM is preferred instead of the huge test separator.

### 5.4. Radioactive Source

Radioactive materials requires careful supervision during execution, operation and disposal. Handling radioactive materials requires permission and experts to meet the given law [162,163]. This means that a MPFM consisting of a radioactive source requires special educated employees, when handling the meter. This is contributing to a higher OPEX and CAPEX due to external employees shipped to the offshore platform for implementing, operating and disposal of the radioactive material.

### 5.5. Calibration

For the MPFM to operate, it needs calibration in the form of input data from time to time. Especially VFMs will need much calibration before start-up to estimate and produce the empirical equations for the software. Contrary to this other MPFM will only need calibration when the accuracy of the data is drifting over time. PT-sensors have a long-term stability and are calibrated with respect to the electric signal from the sensor. Over the time a PT-sensor will drift from the initial zero point offset. By calibration, the zero point offset can be re-adjusted increasing the accuracy. Pressure calibration is done by venting the sensor with ambient air and hereby trimming the offset, so that the zero point is again matching [164]. Gamma-ray source requires special educated personnel and strictly permissions and is therefore, a more comprehensive technology to calibrate. The calibration of a gamma source depends on the half-life time of the source [121]. For a given period stated by the manufacturer, the radioactive source must be calibrated to take account for the loss of intensity of the source. If the half-life time is short, the calibration needs to be performed more frequently compared to a radioactive source with a longer half-life time. The calibration is done by measuring the count-rates of the radiation with respect to each single phase [118,165,166,167].

## 6. Conclusion and Predictions for MPFMs

This review article has presented and discussed the newest trends in the offshore oil and gas industry with respect to multi-phase flow measurements. As stated in this article, the accuracy and reliability of multi-phase flow measurements are essential for allocated production data and model prediction. The value of MPFMs can be of great importance over the entire lifetime of a field, but especially as mature fields turn into brown fields over the production life of the field. Hence, it is essential to monitor production and stage of each well as this can estimate the lifetime and future development of a field. In the latter years of the production life of a well, the focus of production is increasing rather than the exploration of the well. For mature wells, it is extremely important to accurately measure the multi-phase flow, as the water cut will increase and the reservoir pressure will decrease.

Tomography and gamma-ray densitometry have been widely investigated, and commercial meters have been developed. The technologies have been further developed, and some new and promising solutions and prototypes have been tested. Some of the prototypes show measurement errors of less than 0.53%. Commercial MPFMs where illustrated and discussed as well. The most accurate MPFM is a gamma densitometer based on experiments of the prototypes and data sheets of the commercial products. Even though gamma densitometers have the greatest overall accuracy and are able to measure the flow independent of the composition of the phases, the radioactive source is often a considerable limitation. Maintenance and calibration of a radioactive source requires special demands and safety to respect the law. A MPFM solution without a radioactive source is therefore, preferred. Sensor fusion-based DP meters with a software tool (VFM) have also shown progress in recent years. This method is to be preferred in most cases, as this is non-intrusive, non-radioactive and cheap compared to other MPFM technologies. Although the accuracy of VFMs is not as great as gamma densitometers, the solution should be greatly considered due to less maintenance requirements and price.

The number of MPFMs and investigations of accurate and intelligent technologies of multi-phase flow measurement are expected to continuously increase and expand in the coming years because of maturing fields and the focus upon continuous oil production. The overall issue is to design a commercial solution, which can accurately measure the entire GVF and provide accurate and real-time measurements. This should be without compromising safety and the footprint on the respective platform. Other essential qualities for future MPFMs are low maintenance, availability and easy operation. A possible solution could therefore, be further investigation of VFMs, as this has the potential to fulfill the qualities for accurate and reliable multi-phase flow measurements. Eventually VFMs could be combined with e.g., tomography technologies in sensor fusion to obtain even more accurate and reliable meters.

## Figures and Tables

**Table 1:** List of current non-commercial prototypes to monitor multi-phase flow.

Non-Commercial Prototypes				
Source	Technology	Accuracy	Advantages	Disadvantages
[82]	ECT	5% Measurement error.	New improved image reconstruction algorithm. Non-radioactive.	Mostly based on static experimental data.
[83]	AC-based ECT	3% Absolute error.	Non-radioactive.	Only suitable for oil-continuous flows.
[97]	Dual-modal sensor: ECT & ERT	Not informed. Measurements follow the expected trend.	ECT mode when continuous phase is oil (WLR < 40%), ERT mode when continuous phase is water (WLR > 40%). Non-radioactive.	Needs more investigation upon dynamic evaluation.
[100]	MIT & EVT	Single-phase: 0.012% relative error. Multi-phase: 12% relative error.	Robust, low-cost and non-radioactive solution.	Only suitable for water-continuous flows. Needs further improvements w.r.t. accuracy.
[104]	MWT	Not informed. Measurements and simulated values show the same trend.	Designed is intended for the process industry and oil-gas-water flow imaging.	Needs improvements of image quality, when frequency is increased.
[105]	EIT	Not informed. Measurements show the expected trend.	Designed for industrial application.	Needs further development before application (e.g., new measurement system).
[109]	EIT	Capacitance mode: 1.64%. Conductive mode: 2.68%	Fast and robust image restoration algorithm. Simple hardware design.	Only preliminary tests.
[112]	Optical tomography.	Gas inclusion: 0.21% void fraction error. Evans Blue solution: 2.17% void fraction error.	Fast data acquisition.	Limitations of larger GVF than 15%.
[121]	Gamma densitometry.	0.53% measurement error.	Non-intrusive and reliable.	Not tested with gas injections. Contains radioactive source.
[122,123]	Dual modality densitometry.	1% Mean absolute error.	Non-intrusive and able to identify flow regimes.	Contains radioactive source.
[126]	VFM (Orifice plate)	3.52% measurement error.	No radioactive source.	Limitation: fluid composition must remain constant during the measuring period.
[65]	VFM (venturi meter + ECT)	Not informed, but performs good flowrate measurements.	High quality images from the ECT sensor.	Over- and underestimated measurements based on employed model (5 different).
[137]	VFM (venturi meter + ERT)	5% relative error (bubble and slug flow). 10% relative error (annular and stratified flow).	Improved measurement performance. No radioactive source.	Flow regime dependent.

**Table 2:** List of industrial multi-phase flow meters.

Industrial Multi-phase Flow Meters							
**Manufacturer**	Emerson (Roxar) [141,142,143]	Halliburton [144]	Schlumberger [145,146]	Weatherford [147,148,149]	Pietro Fiorentini [150,151,152,153,154,155]	ABB [156]	KROHNE Oil & Gas [157]
**Footprint**	Small	Small	Small	Small	Small	Small	Small
**Radioactive source**	Optional	None	✓	✓	Optional	None	None
**Range**	2600 M/MG: 0–100% WLR 0–85% GVF 2600 MV/MVG: 0–100% WLR 0–100% GVF	0–100% WLR 0–100% GVF	0–100% WLR 0–100% GVF	0–100% WLR 0–100% GVF	Flowatch 3I/HS: 0–100% WLR 0–97% GVF Wetgas meter: 0–100% WLR 0–100% GVF	0–100% WLR 0–100% GVF	0–100% WLR 0–98% GVF
**Technology**	EIT	VFM	Gamma densitometry	VFM (venturi, gamma, sonar array).	Venturi, gamma densitometry, EIT.	VFM (Orifice plate)	Magnetic resonance (MR)
**Accuracy**	2600 M/MG: **Liquid rate**: ±8–10% relative. **Gas rate**: ±8–10% relative. **Water cut**: ±3–5% absolute. 2600 MV/MVG: **Liquid rate**: ±3–5% relative. **Gas rate**: ±6–8% relative. **Water cut**: ±2–4% absolute.		±5%	**Liquid rate**: ±5%. **Gas rate**: ±5%. **Water cut**: ±2%.	Flowatch HS: **Liquid rate**: ±3% relative. **Gas rate**: ±5% relative. **Water cut**: ±2% absolute.	**Gas**: (GVF > 99%): 2% of reading. **Gas**: (90% < GVF > 100%): 3% of reading. **Gas**: (80% < GVF > 90%): 5% of reading. **Gas**: (GVF > 80%): 10% of reading. **Liquid**: (90% < GVF > 100%): 5% of reading.	**Liquid rate**: 3–5% MV. **Gas rate**: 8–10% MV. **Water cut**: 3–5% MV.
**Repeatability**	<2%	±1%	±2%				
**Advantages**	Non-radioactive solutions and add-on equipment available.	No radiation. Simple and cheap solution. Measurements in full range.	No flow calibration plus add-on equipment. Measurements in full range.	Add-on options available. Measurements in full range.	Non-radioactive solutions and add-on equipment available.	No radiation. Simple and cheap solution. Measurements in full range.	No radiation.
**Disadvantages**	Not full GVF depending on model.		Radioactive source.	Radioactive source.	Not full GVF depending on model.	Much calibration before start-up.	Not full GVF.

## Nomenclature

*a*	Cross-section of pipe divided with equal area
*A*	Cross-section area of the pipe
$A_{ori}$	Area of orifice plate
ANN	Artificial neural network
*C*	Capacitance
$C_{d}$	Discharge coefficient
$C_{d,c}$	Discharge coefficient for compressible flows
$C_{d,i}$	Discharge coefficient for incompressible flows
CAPEX	Capital expenditures
$d_{pipe}$	Diameter of pipe
$d_{vc}$	Diameter of pipe at Vena Contracta
*D*	Internal diameter of the pipe
DP	Differential pressure
ECT	Electrical capacitance tomography
EIT	Electrical impedance tomography
EMT	Electromagnetic tomography
ERT	Electrical resistance tomography
EVT	Electromagnetic velocity tomography
$f_{i(x)}$	Volume fraction of phase *x*
FM	Flow meter
$Fr_{g}$	Gas densimetric Froude number
$Fr_{l}$	Liquid densimetric Froude number
*g*	Gravitational force
GVF	Gas volume fraction
LBP	Linear back projection
$m_{g}$	Mass flow rate of the gas
$m_{l}$	Mass flow rate of the liquid
*M*	Mass flow rate
MIT	Magnetic induction tomography
MR	Magnetic resonance
MPFM	Multi-phase Flow Meter
MWT	Microwave tomography
*N*	Number of elements
NIR	Near infra red
OPEX	Operating expenses
P	Pressure
PET	Positron emission tomography
PEPT	Positron emission particle tracking
PT	Pressure and temperature
PW	Produced water
*Q*	Volumetric flow rate
$Q_{x}$	Volumetric flow rate of phase *x*
$U_{sg}$	Superficial gas velocity
$U_{sl}$	Superficial liquid velocity
$v_{g}$	Instantaneous velocity of gas
$v_{i}$	Flow velocity in the *i*-th element
$v_{o}$	Instantaneous velocity of oil
$v_{w}$	Instantaneous velocity of water
*V*	Potential difference
VFM	Virtual Flow Meter
VMS	Virtual Metering System
WLR	Water liquid ratio
$X_{LM}$	Lockhart-Martinelli wet gas parameter
*Y*	Expansion coefficient
*z*	Change in elevation
α	Gas void fraction
β	Water fraction
$\beta_{d}$	Ratio between diameter of pipe and diameter of orifice
χ	Oil fraction
Γ	Electrode surface
ε	Permeability distribution
$\varepsilon_{r(gas)}$	Permittivity of gas
$\varepsilon_{r(oil)}$	Permittivity of oil
$\varepsilon_{r(water)}$	Permittivity of water
ϕ	Electrical potential distribution
$\rho_{g}$	Density of gas
$\rho_{l}$	Density of liquid
$\rho_{o}$	Density of oil
$\rho_{w}$	Density of water
σ	Conductivity distribution
